# Differential Associations between Cortical Thickness and Striatal Dopamine in Treatment-Naïve Adults with ADHD vs. Healthy Controls

**DOI:** 10.3389/fnhum.2017.00421

**Published:** 2017-08-22

**Authors:** Mariya V. Cherkasova, Nazlie Faridi, Kevin F. Casey, Kevin Larcher, Gillian A. O'Driscoll, Lily Hechtman, Ridha Joober, Glen B. Baker, Jennifer Palmer, Alan C. Evans, Alain Dagher, Chawki Benkelfat, Marco Leyton

**Affiliations:** ^1^Division of Neurology, Department of Medicine, University of British Columbia Vancouver, BC, Canada; ^2^Department of Medicine, Stanford University Stanford, CA, United States; ^3^Centre Hospitalier Universitaire Sainte-Justine Montréal, QC, Canada; ^4^Department of Neurology and Neurosurgery, McGill University Montréal, QC, Canada; ^5^Department of Psychology, McGill University Montréal, QC, Canada; ^6^Department of Psychiatry, McGill University Montréal, QC, Canada; ^7^Douglas Institute Montréal, QC, Canada; ^8^Department of Psychiatry, University of Alberta Montréal, QC, Canada; ^9^Ottawa Hospital Ottawa, ON, Canada; ^10^Center for Studies in Behavioral Neurobiology, Concordia University Montréal, QC, Canada

**Keywords:** cortical thickness, dopamine, ADHD, PET, salience network

## Abstract

Alterations in catecholamine signaling and cortical morphology have both been implicated in the pathophysiology of attention deficit/hyperactivity disorder (ADHD). However, possible links between the two remain unstudied. Here, we report exploratory analyses of cortical thickness and its relation to striatal dopamine transmission in treatment-naïve adults with ADHD and matched healthy controls. All participants had one magnetic resonance imaging (MRI) and two [^11^C]raclopride positron emission tomography scans. Associations between frontal cortical thickness and the magnitude of *d*-amphetamine-induced [^11^C]raclopride binding changes were observed that were divergent in the two groups. In the healthy controls, a thicker cortex was associated with less dopamine release; in the ADHD participants the converse was seen. The same divergence was seen for baseline D2/3 receptor availability. In healthy volunteers, lower D2/3 receptor availability was associated with a thicker cortex, while in the ADHD group lower baseline D2/3 receptor availability was associated with a thinner cortex. Individual differences in cortical thickness in these regions correlated with ADHD symptom severity. Together, these findings add to the evidence of associations between dopamine transmission and cortical morphology, and suggest that these relationships are altered in treatment-naïve adults with ADHD.

## Introduction

Attention deficit hyperactivity disorder (ADHD) is thought to arise in part from alterations in catecholamine signaling (Castellanos et al., 1994; Spencer et al., 2005, 2013; Volkow et al., 2009; Cherkasova et al., 2014) and structural abnormalities in multiple cortico-striatal circuits (Nakao et al., 2011; Castellanos and Proal, 2012). These effects might be related. Animal studies suggest that dopamine (DA) has trophic effects during development: D2 receptor agonism promotes neurite growth in cortical neurons (Reinoso et al., 1996), whereas disruption of normal DA transmission diminishes dendritic growth and dendritic length in cortical areas that receive significant DA inputs, such as the prefrontal cortex and anterior cingulate (Kalsbeek et al., 1989; Jones et al., 1996). DA depletion in adult animals likewise results in dystrophic changes to prefrontal dendrites (Wang and Deutch, 2008), and D1 receptor antagonism reduces prefrontal synaptic density (Imai et al., 2004). Some of these effects might begin early in development, and there have been reports that neonatal DA depletion causes reductions in cortical thickness (CT) (Kalsbeek et al., 1987; Alvarez et al., 2002). Conversely, reduced catechol-O-methyltransferase (COMT) activity (which leads to higher cortical DA levels) is associated with increased gray matter volume and cortical thickness in humans and mice (Honea et al., 2009; Cerasa et al., 2010; Witte and Flöel, 2012; Ira et al., 2013; Sannino et al., 2015; Lee and Qiu, 2016); genetically driven reduction in COMT activity likewise results in increased neuronal density in male mice (Sannino et al., 2015). Polymorphisms of the DA receptor 4 gene have also been linked to cortical thickness and prefrontal gray matter volume in ADHD, while findings on DAT1 genotypes have been inconclusive (Durston et al., 2005; Shaw et al., 2007; Fernández-Jaén et al., 2015, 2016). Little is known about relationships between brain morphology and *in-vivo* measures of DA transmission in humans. However, recent studies suggest that, in healthy volunteers, gray matter density and volume are related to D2 receptor availability (measured with [^18^F]fallypride) in the midbrain, striatum, thalamus, amygdala, and diverse cortical areas (Woodward et al., 2009), while striatal DA release is related to CT (Casey et al., 2013; Jaworska et al., 2017) and cortico-striatal anatomical connectivity (Tziortzi et al., 2014).

These associations might have functional significance, as other evidence suggests cortical regulation of striatal DA transmission. Ablation of prefrontal DA terminals in rats can increase striatal DA transmission (Pycock et al., 1980). Similarly, in healthy humans increased metabolic activity in prefrontal cortex is associated with smaller amphetamine-induced striatal DA release (Goldstein et al., 2007), while transcranial magnetic stimulation, which is thought to inhibit the prefrontal cortex, elicits striatal DA release (Strafella et al., 2001, 2003, 2005; Ohnishi et al., 2004; Ko et al., 2014).

Associations between DA signaling and cortical structure and function might be disturbed in people with ADHD (Sonuga-Barke, 2005; Petrovic and Castellanos, 2016), but this has yet to be investigated directly. To explore hypothesized associations between striatal DA transmission and CT, we analyzed the data from a previously reported study in treatment-naïve adult men with ADHD vs. healthy controls (Cherkasova et al., 2014). We focused on changes in [^11^C]raclopride binding in response to a *d*-amphetamine challenge, which are proportional to DA efflux (Breier et al., 1997), and [^11^C]raclopride binding potential values, which measure the availability of striatal D2/3 receptors. Based on our previous findings in healthy volunteers (Casey et al., 2013), we hypothesized that CT would be related to indices of striatal DA transmission and that these associations would be altered in participants with ADHD.

## Methods

### Participants

The participants were 15 adult men with ADHD (5 combined, 10 predominantly inattentive subtype) and 18 male healthy controls, all of whom took part in a previously reported study of DA function in treatment-naïve ADHD (Cherkasova et al., 2014). In brief, the diagnosis of ADHD (DSM-IV-TR) was ascertained by a research psychiatrist (CB, LH, RJ), and ADHD symptoms were measured in both groups using the Conners' Adult ADHD Rating Scale (CAARS) (Conners et al., 1999) (Table 1). Participants were free from any current or past Axis I disorder other than ADHD (Structured Clinical Interview for DSM-IV Axis I disorders First et al., 1996), except two ADHD participants who reported a single mild depressive episode occurring ≥2 years in the past. Other exclusion criteria were: a first-degree relative with a history of substance dependence; current use of psychotropic medications; Beck Depression Inventory (BDI) (Beck and Steer, 1993) score > 13, estimated IQ < 80; a neurological history; reported history of serious head injury; history of any physical disorder (e.g., cardiovascular) contradictory to participation; and a positive toxicology screen as per the Triage Drugs of Abuse urine test (Biosite Inc., San Diego). Controls were additionally excluded for a reported ADHD diagnosis in a first-degree relative. All ADHD participants were stimulant treatment-naïve except one, who 2 years prior to his participation underwent a 6-month methylphenidate trial. Excluding his data did not change the results. Lifetime stimulant exposure did not exceed two uses for any other participants.

**Table 1:** Sample characteristics.

	**Controls (*n* = 18)**	**ADHD (*n* = 15)**	**Test statistic (df)**	***P***
Age (SD)	25.44 (6.77)	29.87 (8.65)	*U*_(33)_ = 99.50	0.20
Estimated full scale IQ (SD)	116.83 (16.07)	107.13 (12.78)	*U*_(27)_ = 51.00	0.06
Abbreviated WAIS-III^1^	124.25 (14.70)	109.44 (15.16)	*U*_(17)_ = 17.00	0.07
Abbreviated WAIS-R^2^	102.00 (1.63)	103.67 (8.12)	*U*_(10)_ = 11.50	0.91
Years of education (SD)	17.11 (3.32)	16.20 (3.63)	*t*_(31)_ = 0.75	0.46
CAARS *t*-scores (SD)				
Inattention/memory problems	43.77 (7.41)	74.00 (10.49)	*t*_(26)_ = 8.67	<0.0005^*^
Hyperactivity/restlessness	43.76 (6.08)	62.27 (12.93)	*t*_(21.79)_ = 4.82	<0.0005^*^
Impulsivity/emotional lability	42.92 (9.42)	58.53 (11.28)	*t*_(26)_ = 3.94	0.001^*^
Problems with self-concept	43.08 (5.89)	63.07 (7.64)	*t*_(26)_ = 7.66	<0.0005^*^
DSM-IV inattention	48.73 (12.49)	84.4 (8.73)	*t*_(26)_ = 8.85	<0.0005^*^
DSM-IV hyperactivity	44.69 (8.54)	68.13 (14.48)	*t*_(26)_ = 5.11	<0.0005^*^
DSM-IV total	46.69 (11.64)	81.47 (10.30)	*t*_(26)_ = 8.39	<0.0005^*^
ADHD index	42.00 (8.45)	66.86 (8.74)	*t*_(26)_ = 7.63	<0.0005^*^
BDI at intake (SD)	1.53 (2.00)	6.04 (3.86)	*U*_(31)_ = 23.50	<0.0005^*^
Recreational drug use history				
Stimulants: No. of lifetime uses (SD)	0.06 (0.24)	0.36 (0.74)	*U*_(32)_ = 105.00	0.17
Marijuana: No. of lifetime uses (SD)	18.00 (33.08)	49.27 (90.07)	*U*_(32)_ = 119.50	0.22
Nicotine: No. of lifetime uses (SD)	1359.06 (3677.02)	1614.13 (5171.99)	*U*_(33)_ = 128.00	0.81
No. of smokers	1	1		

Group Differences:

* *p ≤ 0.05*.

1 *Wechsler Adult Intelligence Scale—Revised (WAIS-R) (n = 9) (Reynolds et al., 1983)*.

2 *Wechsler Adult Intelligence Scale—III (WAIS-III) (n = 9) (Pilgrim et al., 1999)*.

The study was carried out in accordance with the Declaration of Helsinki and was approved by the Research Ethics Board of the Montreal Neurological Institute. All participants gave written informed consent.

### Procedure

Participants first underwent an assessment to determine their eligibility to take part in the study based on the criteria described above. Eligible participants underwent two double-blind, randomized, and counterbalanced [^11^C]raclopride PET scans, one following a lactose placebo and the other following 0.3 mg/kg p.o. of *d*-amphetamine; capsule administration occurred 60 min before tracer injection. PET scans occurred at least 3 days apart. The mean interval between the two PET scans was longer the ADHD participants (23.1 days, SD = 25.1) than controls (8.4 days, SD = 5.5) [U_(32)_ = 70, *p* =0.03]. The longer interval for the ADHD group resulted from several outliers, and the median interval did not differ between groups (ADHD: 15 days; controls: 7 days, *p* = 0.30). Before each scan, participants were asked to abstain from food, caffeine and smoking for 4 h and from alcohol for 24 h. A structural MRI was obtained on a separate day. The average interval between the PET and MR scans was 22.0 (SD = 21.9) for the ADHD participants and 21.8 (SD = 35.1) for the controls (*p* = 0.52). Toxicology screening occurred on the initial screening interview and prior to both PET scans.

### Neuroimaging

Participants were scanned on a Siemens ECAT HR + PET scanner (CTI/Siemens, Knoxville, Tennessee) with lead septa removed (63-slice coverage), with a maximum resolution 4.2-mm, full width at half maximum (FWHM) in the center of the field of view. Attenuation correction was performed using a 12-min ^68^Ga transmission scan immediately prior to tracer injection. The emission scan started simultaneously with the injection of [^11^C]raclopride, as an i.v. bolus, and data were acquired for 60 min in 26 time frames of progressively longer duration. Vital signs were monitored and blood samples for plasma amphetamine collected just prior to capsule administration, at the time of tracer administration, mid-scan, and at the end of scan.

High-resolution (1 mm) T1-weighted magnetic resonance images (MRI) were obtained on a 1.5-Tesla Siemens scanner, using gradient echo pulse sequence (*TR* = 22 ms, *TE* = 9.2 ms, flip angle = 30°, *FOV* = 256 mm, and matrix 256 × 256).

### Analyses

#### PET

As previously (Casey et al., 2013; Cherkasova et al., 2014), the PET images were reconstructed using a 6-mm full-width at half-maximum Hanning filter and corrected for motion (Costes et al., 2009). Summed radioactivity PET images for each participant were co-registered with the individual's MRI. MRIs were first corrected for image intensity non-uniformity (Sled et al., 1998) and transformed into the Montreal Neurological Institute (MNI) stereotaxic space using automated feature matching to the MNI305 template (Collins et al., 1994). The MRI transformation algorithm was then concatenated with the PET to MNI co-registration information to transform the summed PET images into the MNI space; all subsequent analyses were carried out in the MNI space. Parametric images were generated by calculating [^11^C]raclopride binding potential values (BP_ND_) (Innis et al., 2007) at each voxel using a simplified reference tissue compartmental model (SRTM) with cerebellum as the reference tissue (Lammertsma and Hume, 1996; Gunn et al., 1997). BP_ND_ is a function of the estimated concentration of available D2/3 receptors (B_Avail_), the dissociation constant of the radiotracer from the receptors (K_D_) and the free fraction of the non-specifically bound tracer in the brain (F_ND_): BP_ND_ = F_ND_^*^(B_Avail_/K_D_).

Mean BP_ND_-values from each individual parametric image were extracted from regions of interest (ROIs) defined on each individual's MRI. The ROIs were based on the functional organization of the striatum (Martinez et al., 2003): limbic (LST, includes ventral striatum), associative (AST, includes pre-commissural dorsal caudate, pre-commissural dorsal putamen, post-commissural caudate), and sensorimotor (SMST, includes post-commissural putamen). Mean BP_ND_-values were corrected for partial volume effects (Aston et al., 2002). ΔBP_ND_-values were calculated as (BP_ND placebo_ – BP_ND *d*−amph_)/BP_ND placebo_ × 100. Prior to analysis of associations of these values with CT, outliers in the distribution were winsorized (replaced by the value of their nearest neighbor (Dixon and Yuen, 1974)); two participants (one ADHD and one control) had outlier ΔBP_ND_-values in SMST and AST.

### Cortical thickness

Native T1 weighted MRIs were processed through the CIVET automated pipeline (version 1.1.11, in-house software developed in the lab of Alan C. Evans, Montreal Neurological Institute) (Benedetti et al., 2006). CIVET outputs cortical surfaces and CT measurements at 40,962 vertices per hemisphere (Collins et al., 1995; MacDonald et al., 2000; Kim et al., 2005; Benedetti et al., 2006; Lyttelton et al., 2007).

### Statistical analysis

Statistical analyses were implemented using SurfStat, a statistical toolbox created in MATLAB by Dr. Keith Worsley (http://www.math.mcgill.ca/keith/surfstat/). Absolute native-space CT values (blurred to 20 mm) for each subject were entered into general linear models (GLM) predicting CT at each vertex from BP_ND_ and ΔBP_ND_ in the three striatal ROIs outlined above (LST, AST, SMST), Group membership (ADHD vs. Control), and the interaction of these terms, with total brain volume as a covariate:

$$\begin{array}{l} CT=\beta_{1}+\beta_{2}BP_{ND}+\beta_{3}Group+\beta_{4}{BP_{ND}}^{*}Group \end{array}$$

Total brain volume, age and BDI scores were all considered as potential covariates. Their inclusion was based on whether adding each covariate to the model significantly improved its fit. This was evaluated using the SurfStatF. Only total brain volume significantly improved the model's fit, so this was the only covariate used.

The effect of group membership on the relationship of CT with BP_ND_ and ΔBP_ND_ was examined by testing the significance of the *BP*_ND_^*^Group and Δ*BP*_ND_^*^Group interaction terms: their significance indicated that regression slopes describing the relationship between CT and BP_ND_/ΔBP_ND_ differed significantly between the groups. Vertex and cluster significance was determined using Random Field Theory (RFT) for non-isotropic images (Worsley et al., 1999) with significance threshold of *p* = 0.05, corrected. For each cluster where the interaction was significant, mean CT was computed for each participant, and its relationship with BP_ND_/ΔBP_ND_ characterized in each group using bivariate correlations (using partial correlations with total brain volume as a covariate did not change the pattern of results). The significance thresholds for these latter correlations were uncorrected, as RFT correction was not possible in these analyses.

To test the robustness of these correlations, confirmatory *post-hoc* analyses were performed predicting vertex-wise CT and from BP_ND_/ΔBP_ND_ in each group using the following GLM:

$$\begin{array}{l} CT=\beta_{1}+\beta_{2}TotalBrainVolume+\beta_{3}BP_{ND} \end{array}$$

This was done to identify associations that, despite contributing to an interaction, are weak and do not survive the RFT threshold on their own. This could occur (a) if an interaction is primarily driven by a strong association in one group and a weak opposite association in the other group (significant uncorrected, but no longer significant using the RFT threshold); (b) if an interaction is driven by two such weak associations, in which case the meaningfulness of the interaction is questionable. If a cluster showing a significant Group^*^BP_ND_/ΔBP_ND_ interaction remained significant in the confirmatory group-wise analysis, it was considered a valid finding.

Because possible group differences in CT between ADHD and Control groups, which have been reported previously (Bush et al., 2008; Proal et al., 2011; Duerden et al., 2012), could bear on the relationship between CT and BP_ND_/ΔBP_ND_, we also compared CT between ADHD and Control groups using both vertex-wise and a region of interest (ROI) analyses. The latter was performed because the significance threshold of the vertex-wise analysis (based on RFT) may be too conservative to detect significant differences in our relatively small sample. The vertex-wise analysis used the following GLM:

$$\begin{array}{l} CT=\beta_{1}+\beta_{2}Group \end{array}$$

We did not covary out total brain volume in this analysis. We considered age as a covariate, but it did not improve the fit of the model. For the ROI analysis, mean CT-values for each participant were extracted from the following ROIs defined on the average cortical surface using the automated anatomical labeling set (Tzourio-Mazoyer et al., 2002): frontal (labels 1–28), insula (labels 29 and 30), limbic (labels 31–40), occipital (labels 43–56), parietal (labels 57–70), and temporal (labels 79–90). Between-group comparisons of these values were then made.

## Results

### Participants

The ADHD group did not differ significantly from Controls on any of the demographic variables. Estimated IQ was marginally higher in the control group (*p* = 0.06) (Table 1). Although no participant was clinically depressed, the ADHD BDI scores at intake were higher than those of Controls (*p*_s_ < 0.0005). Because of this group difference and a significant correlation between BDI and ΔBP_ND_ in ADHD participants only (*r* = −0.68 *p* = 0.007), BDI was considered as a covariate, but it was not used in the final models since it did not improve the fit.

### Baseline D2/3 and *d*-amphetamine-induced change in D2/3 binding

In the healthy controls, there was a significant *d*-amphetamine-induced decrease in BP_ND_ in LST [*t*_(17)_ = 5.32, *p* < 0.0005], but not in AST or SMST (*p*_*s*_ > 0.1). In ADHD subjects, there were significant BP_ND_ decreases in all three ROIs [AST: *t*_(14)_ = 2.15, *p* = 0.05; SMST: *t*_(14)_ = 3.24, *p* = 0.006; LST: *t*_(14)_ = 2.36, *p* = 0.03), and these effects were significantly greater than those seen in the controls within both the AST and SMST [Group × ROI interaction: *F*_(1.36, 39.70)_ = 4.07; *p* = 0.04; AST: *F*_(1, 30)_ = 4.24, *p* = 0.05; SMST: *F*_(1, 30)_ = 4.73, *p* = 0.04]. See Cherkasova et al. (2014) for more detail.

BP_ND_-values on the placebo and *d*-amphetamine scans and ΔBP_ND_s are given in Table 2. As reported previously, the groups did not differ significantly on baseline BP_ND_ (*p*_s_ ≥ 0.07).

**Table 2:** D2/3 binding potential (BP_ND_) and ΔBP_ND_ in striatal ROIs.

	**Controls**				**ADHD**			
	**Placebo**	***d*-amph**	**Placebo vs. *d*-amph**	**ΔBP_ND_**	**Placebo**	***d*-amph**	**Placebo vs. *d*-amph**	**ΔBP_ND_**
AST (SD)	3.00 (0.41)	3.00 (0.49)	*t*_(17)_ = −0.04; *p* = 0.97	–0.57%	3.25 (0.35)	3.04 (0.43)	*t*_(14)_ = 2.15; *p* < 0.05	6.08%
SMST (SD)	3.53 (0.47)	3.41 (0.54)	*t*_(17)_ = 1.37; *p* = 0.19	3.25%	3.76 (0.49)	3.37 (0.48)	*t*_(14)_ = 3.24; *p* < 0.006	9.68%
LST (SD)	3.26 (0.39)	2.86 (0.47)	*t*_(17)_ = 5.32; *p* < 0.0005	12.34%	3.16 (0.53)	2.85 (0.43)	*t*_(14)_ = 2.36; *p* < 0.03	8.62%

*BP_ND_-values for Control and ADHD groups on placebo and d-amph. AST, associative ROI; SMST, sensorimotor ROI; LST, limbic ROI*.

### Group differences in cortical thickness

We examined whether cortical thickness differed between groups using vertex wise and ROI analyses. Vertex-wise analysis did not reveal any significant clusters. ROI analyses found greater CT in controls than ADHD participants in the right frontal lobe [*t*_(31)_ = 2.04, *p* = 0.05], left insula [*t*_(31)_ = 2.19, *p* = 0.04], and left and right temporal lobes [left: *t*_(31)_ = 2.26, *p* = 0.03; right: *t*_(31)_ = 2.52, *p* = 0.02] but these trends did not meet family-wise correction for multiple comparison (*p* < 0.004).

### Associations between *d*-amphetamine-induced ΔBP_ND_ and cortical thickness

**Figure 1:**
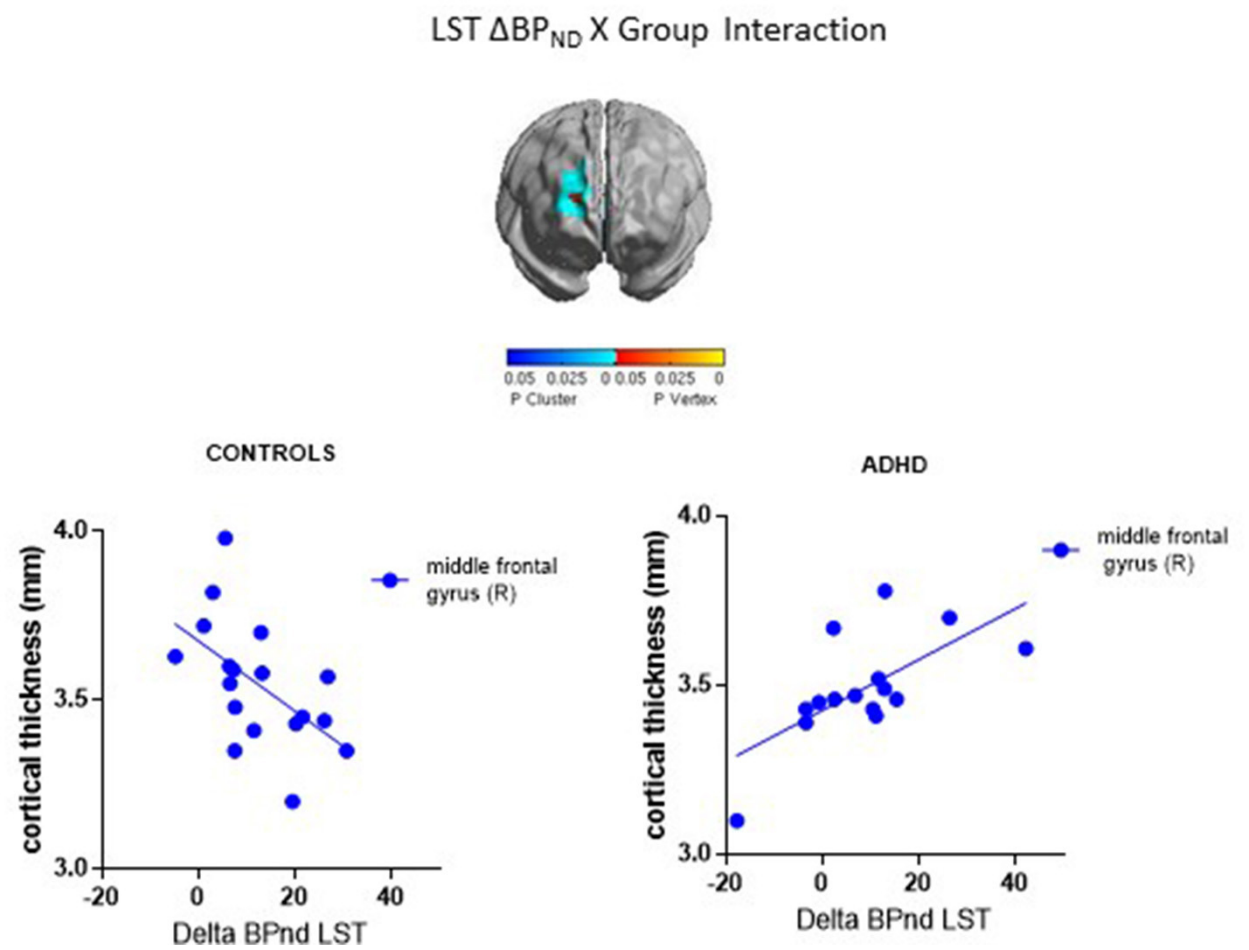
Loci of association of ΔBP_ND_ with cortical thickness (CT).

ΔBP_ND_ values in the LST interacted with Group to predict cortical thickness in a cluster in the right middle frontal gyrus (*p* < 0.00005, RFT-corrected) (Figure 1, Table 3). Group-wise correlations to characterize the relationship between LST ΔBP_ND_ and mean CT in this cluster showed that in the healthy controls, smaller LST ΔBP_ND_ values were associated with a thicker middle frontal gyrus [*r*_(18)_ = −0.55; *p* = 0.02, uncorrected), whereas the converse was seen in the ADHD participants [*r*_(15)_ = 0.66; *p* = 0.008, uncorrected]. Separate vertex-wise analyses in each group using RFT thresholding showed that the interaction was most prominently driven by a negative association between LST ΔBP_ND_ and CT in the control subjects [*r*_(18)_ = −0.57, *p* = 0.001, corrected; Table 4). There was also a significant interaction of SMST ΔBP_ND_ values with Group on CT in the left supplementary motor area (SMA) (Figure 1, Table 3). As in the LST, this interaction reflected divergent associations in the controls and ADHD participants: a trend for a negative association between ΔBP_ND_ and mean CT was seen in the healthy volunteers [*r*_(18)_ = −0.36; *p* = 0.14, uncorrected] while in ADHD participants the association was positive [*r*_(15)_ = 0.64; *p* = 0.01, uncorrected; Table 3]. The SMA cluster did not emerge as significant in group-wise confirmatory analysis. The pattern of associations of ΔBP_ND_ with mean CT was not altered by including total brain volume as a covariate in these correlation analyses (data not shown). Together, the analyses identified evidence of converse associations between CT and striatal DA release in healthy controls (negative correlations) and volunteers with ADHD (positive correlations).

**Table 3:** *d*-Amphetamine induced change in BP_ND_ in relation to cortical thickness.

**Cluster**	**No. of vertices**	**No. of resels**	***x***	***y***	***z***	***t***	***p***	***r* (Controls, ADHD)**
**LIMBIC STRIATUM** Δ**BP**_ND_ **X GROUP INTERACTION**								
Right middle frontal gyrus	704	4.51	27.78	53.04	17.51	4.72	<0.0005	−0.55^*^, 0.66^*^
**Confirmatory** ***Post-hoc*** **Analysis: Controls**								
Right middle frontal gyrus	304	1.74	26.24	51.24	51.72	4.64	0.001	−0.57^*^
**Confirmatory** ***Post-hoc*** **Analysis: ADHD**								
–	–	–	–	–	–	–	–	–
**SENSORIMOTOR STRIATUM** Δ**BP**_ND_ **X GROUP INTERACTION**								
Left supplementary motor area	223	1.23	−11.71	−2.74	71.73	4.48	0.002	−0.37, 0.79
**Confirmatory** ***Post-hoc*** **Analysis: Controls**								
–	–	–	–	–	–	–	–	–
**Confirmatory** ***Post-hoc*** **Analysis: ADHD**								
–	–	–	–	–	–	–	–	–

*Clusters showing a significant ΔBP_ND_ by Group interaction followed by clusters showing significant associations of CT with BP_ND_ in within-group confirmatory post-hoc analyses*.

All p-values for cluster thresholds are RFT-corrected. P-values for correlations between mean cortical thickness in a cluster and BP_ND_/ΔBP_ND_ are not RFT-corrected (

* *p < 0.05, uncorrected)*.

**Table 4:** Baseline BP_ND_ in relation to cortical thickness.

**Cluster**	**No. of vertices**	**No. of resels**	***x***	***y***	***z***	***t***	***p***	***r* (Controls, ADHD)**
**SENSORIMOTOR STRIATUM BP**_**ND**_ **X GROUP INTERACTION**								
Right cerebellum	131	2.00	38.44	−39.96	−21.96	5.72	0.001	−0.58^*^, 0.81^*^
Right anterior cingulate	334	1.67	8.08	8.81	40.18	3.90	0.003	−0.42, 0.85^*^
Left cuneus	371	1.65	−9.74	−68.81	12.86	4.56	0.004	−0.55^*^, 0.75^*^
Left posterior insula	210	1.00	−33.41	−30.63	18.41	4.69	0.03	−0.44, 0.84^*^
**Confirmatory** ***Post-hoc*** **Analysis: Controls**								
–	–	–	–	–	–	–	–	–
**Confirmatory** ***Post-hoc*** **Analysis: ADHD**								
Right cerebellum	65	0.99	39.99	−40.13	−21.51	4.83	0.03	0.82^*^
Right anterior cingulate	463	4.19	8.97	−2.43	40.36	4.86	0.0001	0.87^*^
Left lingual gyrus	214	1.74	−15.05	−60.07	−3.86	4.87	0.006	0.85^*^
Left posterior insula	181	1.02	−32.80	−28.51	20.69	6.09	0.03	0.85^*^
Right insula^‡^	692	12.06	37.20	−6.93	11.14	4.72	<0.00005	0.91^*^
Left insula^‡^	345	2.55	−36.87	3.80	7.27	4.86	0.001	0.85^*^
**ASSOCIATIVE STRIATUM BP**_**ND**_ **X GROUP INTERACTION**								
Left cerebellum	464	2.11	−28.66	−56.35	−8.68	4.33	0.001	−0.34, 0.91^*^
Left cuneus	455	2.08	−9.60	−69.41	10.88	5.83	0.001	−0.58^*^, 0.85^*^
Right cerebellum	82	1.24	40.62	−36.01	−22.96	4.16	0.01	−0.47, 0.76^*^
Right dorsal anterior cingulate	338	1.15	8.67	16.70	39.65	3.99	0.02	−0.48^*^, 0.87^*^
**Confirmatory** ***Post-hoc*** **Analysis: Controls**								
–	–	–	–	–	–	–	–	–
**Confirmatory** ***Post-hoc*** **Analysis: ADHD**								
Left cerebellum	393	2.27	−27.30	−50.36	−12.05	5.31	0.002	0.92^*^
Left cuneus	239	1.62	−3.87	−82.48	12.53	6.21	0.007	0.87^*^
Right dorsal anterior cingulate	319	2.20	9.07	20.45	33.71	5.26	0.002	0.88^*^
Right anterior insula^‡^	213	8.76	37.16	8.27	−6.52	5.63	<0.00005	0.92^*^
Left ventral anterior cingulate^‡^	180	1.91	−7.37	−8.29	42.84	5.60	0.004	0.85^*^
**LIMBIC STRIATUM BP**_**ND**_ **X GROUP INTERACTION**								
Right dorsal posterior cingulate	201	1.24	12.00	−23.35	42.16	4.53	0.009	−0.60^*^, 0.68^*^
Left dorsal posterior cingulate	229	1.17	−9.93	−26.32	44.53	3.74	0.02	−0.47^*^, 0.73^*^
Right supramarginal gyrus	138	1.00	52.34	−53.51	28.66	4.25	0.03	−0.72^*^, 0.53^*^
**Confirmatory** ***Post-hoc*** **Analysis: Controls**								
–	–	–	–	–	–	–	–	–
**Confirmatory** ***Post-hoc*** **Analysis: ADHD**								
–	–	–	–	–	–	–	–	–

*Clusters showing a significant BP_ND_ by Group interaction followed by clusters showing significant associations of CT with BP_ND_ in within-group confirmatory post-hoc analyses*.

‡ *Clusters that did not show a significant interaction with Group, but emerged as showing significant association between cortical thickness and BP_ND_ in the ADHD group post-hoc analysis*.

All p-values for cluster thresholds are RFT-corrected. P-values for correlations between mean cortical thickness in a cluster and BP_ND_/ΔBP_ND_ are not RFT-corrected (

* *p < 0.05, uncorrected)*.

### Associations between baseline BP_ND_ and cortical thickness

**Figure 2:**
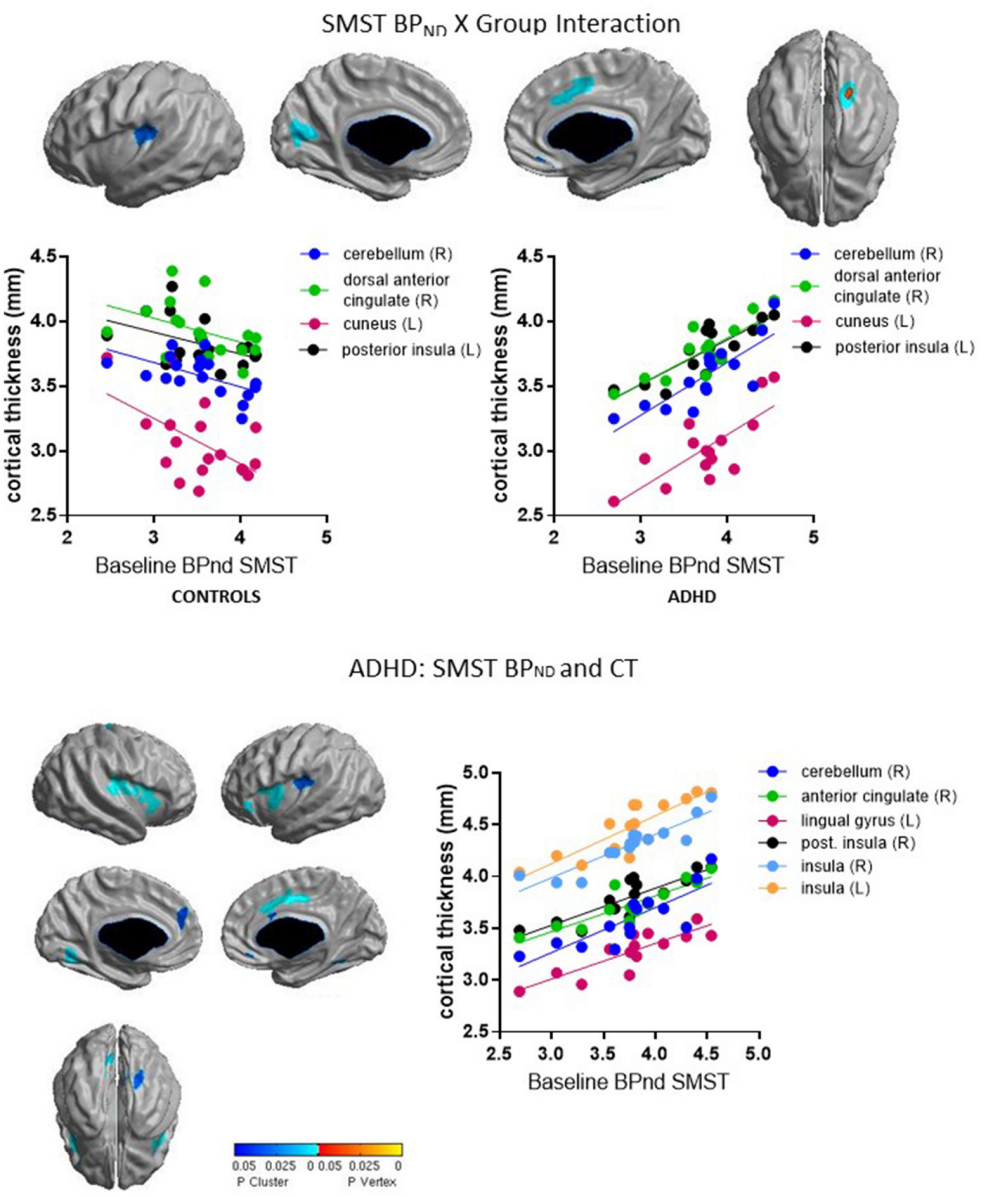
Loci of association of baseline BP_ND_ in SMST with cortical thickness (CT).

**Figure 3:**
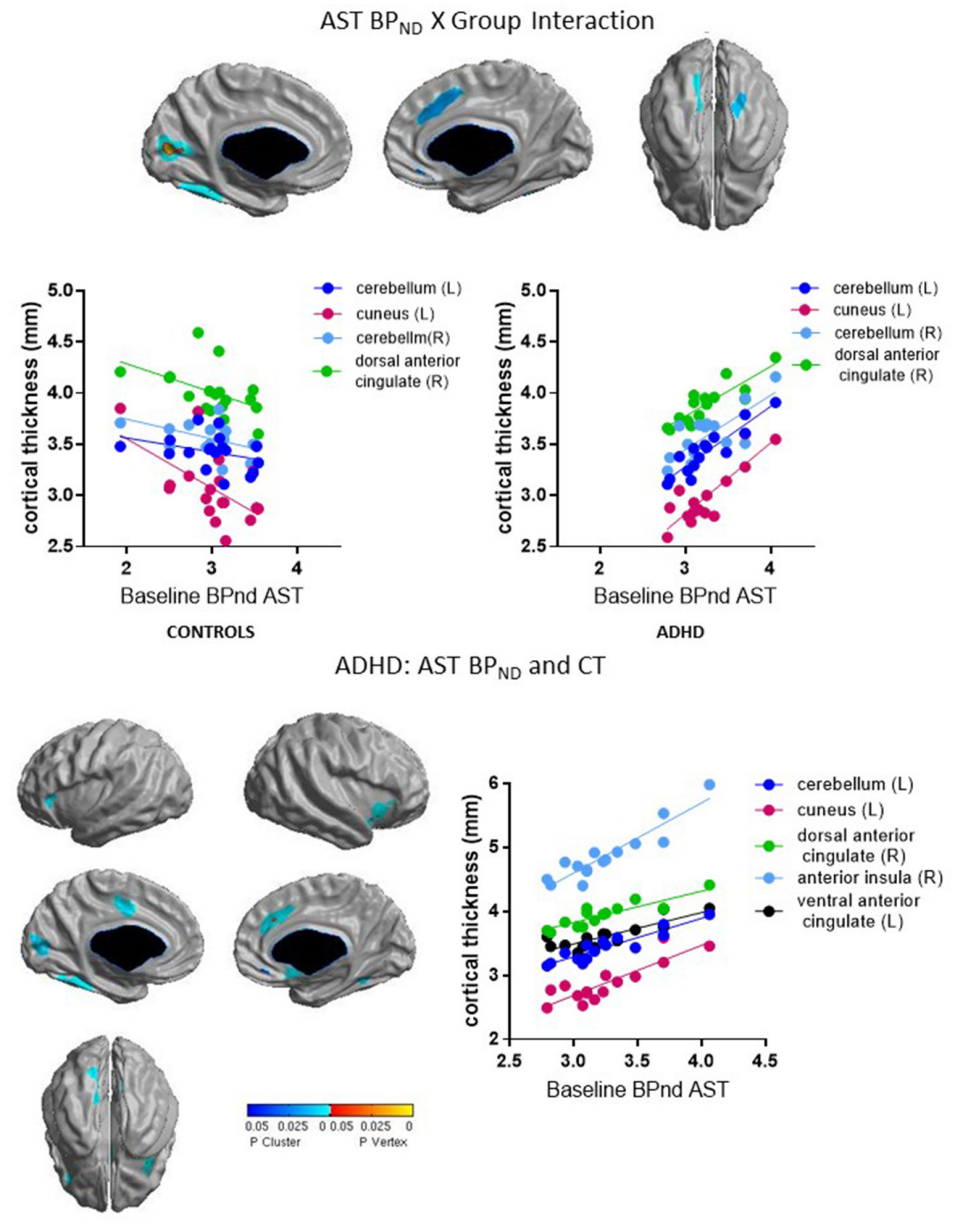
Loci of association of baseline BP_ND_ in AST with cortical thickness (CT).

Group interacted with BP_ND_ in the SMST and AST to predict CT in overlapping regions, such that higher BP_ND_ was associated with thicker cortex in ADHD but with thinner cortex in controls (Figures 2, 3, Table 4). In the SMST, the interaction was significant in right dorsal anterior cingulate, right cerebellum, left cuneus, and left posterior insula (*p*_*s*_ ≤ 0.03, corrected). In the AST, the interaction was significant in right dorsal anterior cingulate, right cerebellum, left cerebellum and left cuneus (*p*_s_ ≤ 0.02, corrected; Table 4). As with the ΔBP_ND_-values, these interactions reflected opposite associations in the two groups. In the healthy controls, BP_ND_ was inversely associated with CT [SMST: right cerebellum *r*_(18)_ = −0.58, *p* = 0.01; right anterior cingulate *r*_(18)_ = −0.43, *p* = 0.08; left cuneus *r*_(18)_ = − 0.55, *p* = 0.02; left posterior insula *r*_(18)_ = −0.44, *p* = 0.07; AST: left cerebellum *r*_(18)_ = −0.34, *p* = 0.17; right cerebellum *r*_(18)_ = −0.47, *p* = 0.051; left cuneus *r*_(18)_ = −0.58, *p* = 0.01; right dorsal anterior cingulate *r*_(18)_ = −0.48, *p* = 0.04, all *p*_*s*_ uncorrected). Conversely, CT in these clusters was positively associated with BP_ND_ in the ADHD subjects [SMST BP_ND_: *r*_s(15)_ ≥ 0.75; AST BP_ND_: *r*_s(15)_ ≥ 0.76]. Separate vertex-wise analyses in each group showed that the interactions were primarily driven by positive associations between BP_ND_ and CT in the ADHD group. In these confirmatory analyses, clusters in the right (SMST) and left (AST) cerebellum (for AST and SMST BP_ND_, respectively), right anterior cingulate (SMST and AST), left posterior insula (SMST), and left occipital clusters (SMST and AST) and left posterior insula (SMST) remained significant in the ADHD group [*r*_s(15)_ ≥ 0.82, *p* ≤ 0.03, corrected; Figures 2, 3, Table 4]; no clusters remained significant in the control group. BP_ND_ in the ADHD group also showed highly significant associations with cortical thickness in the insula (particularly on the right) [*r*_s(15)_ ≥ 0.85, *p*_s_ ≤ 0.001, corrected]. Because of the size of these clusters and the strength of the associations, we report them in Table 4.

For BP_ND_ in the LST, the interaction was significant in three clusters located in the right posterior cingulate, left posterior cingulate and supramarginal gyrus (*p*_s_ ≤ 0.03, corrected). In these clusters BP_ND_-values were inversely associated with CT in the healthy controls [right posterior cingulate: *r*_(18)_ = −0.60; *p* = 0.009; left posterior cingulate: *r*_(18)_ = −0.47; *p* = 0.05; right supramarginal gyrus: *r*_(18)_ = −0.72; *p* = 0.001, uncorrected] but positively associated in ADHD [right posterior cingulate: *r*_(15)_ = 0.68; *p* = 0.005; left posterior cingulate: *r*_(15)_ = 0.74; *p* = 0.002; right supramarginal gyrus: *r*_(15)_ = 0.53; *p* = 0.04, uncorrected; Table 4]. However, these clusters did not emerge as significant in the confirmatory vertex-wise analyses in each group using RFT thresholding. As for ΔBP_ND_, the pattern of associations of mean CT with BP_ND_ was not altered by including total brain volume as a covariate (data not shown).

To summarize, we found divergent associations in healthy volunteers (negative correlations) and ADHD subjects (positive correlations) between baseline BP_ND_ in SMST and AST and CT in anterior cingulate, cerebellar, and occipital regions.

### Clusters associated with DA function and ADHD symptoms

As a final analysis, we examined whether cortical thickness in clusters that were associated with binding measures co-varied with symptom severity. Due to the number of clusters showing significant associations with baseline BP_ND_, we used a principal component analysis (PCA) to reduce the number of variables. We entered the clusters showing significant associations with BP_ND_ in ADHD subjects (including both the clusters where we found a significant interaction with Group and the clusters that emerged in the *post-hoc* analysis) into a PCA with an oblique rotation; Kaiser-Meyer-Olkin (KMO) value of 0.74 confirmed sampling adequacy. The PCA yielded a single factor with the eigenvalue of 8.73 and accounting for 79.3% of the variance; factor loadings ranged from 0.84 to 0.93. We then asked whether scores on this factor indexing cortical thickness associated with baseline BP_ND_ were inversely related to ADHD symptom severity. As the measure of ADHD symptom severity, we used the ADHD Index scores of the CAARS. The ADHD Index is a global composite scale of this instrument containing the items that best distinguish ADHD adults from non-clinical adults. In the ADHD subjects, higher ADHD symptom scores were associated with lower scores on the cortical thickness factor, indicating thinner cortex [*r*_(15)_ = −0.54, *p* = 0.04]. In the healthy controls, factor scores did not correlate with ADHD Index [*r*_(13)_ = 0.05; *p* = 0.86]. Cortical thickness in the middle frontal gyrus cluster (that was associated with ΔBP_ND_ in controls) was not significantly correlated with ADHD index. In summary, regional cortical thickness associated with higher BP_ND_ correlated with severity of ADHD symptoms in ADHD subjects.

## Discussion

We report exploratory analyses of the relation between striatal DA function and cortical thickness. Two main findings were observed. First, in the healthy control group, individual differences in *d*-amphetamine-induced DA release in the LST were negatively related to CT. In treatment-naïve adults with ADHD, the opposite was observed. Second, in the ADHD adults, baseline striatal D2/3 receptor availability in sensorimotor and associative striatal sub-regions was strongly and positively associated with CT, most prominently in the anterior cingulate and insula. In comparison, in healthy controls, the relation between CT with D2/3 binding trended in the opposite direction. Finally, thinner cortex in the regions where cortical thickness was associated with baseline D2/3 availability was associated with more severe symptoms in the ADHD subjects.

A negative association between striatal DA release and CT in healthy volunteers has been reported by us previously (Casey et al., 2013; Jaworska et al., 2017). In the context of existing evidence of prefrontal regulation of striatal DA (Bertolino et al., 2000; Strafella et al., 2001; Meyer-Lindenberg et al., 2002; Volkow et al., 2007), we interpret this inverse association to potentially reflect more effective regulation of striatal DA in those with thicker prefrontal cortex (PFC). Though work in rodents shows that PFC projections to midbrain play a central role in regulating firing patterns of DA neurons (Sesack and Carr, 2002), PFC projections to the DA-ergic midbrain are sparse in primates (Frankle et al., 2006). Potentially more important, then, are PFC projections to the striatum. For example, the PFC drives population activity of DA neurons via its excitatory projections to nucleus accumbens by weakening ventral pallidal inhibition of DA neuron activity (Grace et al., 2007); this population activity determines striatal DA tone, which has been proposed to influence phasic DA release likely measured by raclopride displacement (Grace, 2001; Grace et al., 2007). The absence of negative associations between CT and DA release may, among other possibilities, signify a disruption of typical prefrontal modulation of DA signaling. Dopaminergic modulation of cortical and limbic inputs to the striatum is important for control of goal-directed behavior (Grace et al., 2007), and disruption of these modulatory mechanisms could result in difficulties with control of motivated behavior seen in ADHD. Another interpretation could be that abnormalities in DA transmission result in alterations to cortical structure, possibly owing to the trophic effects of DA during development reported in animal studies (Kalsbeek et al., 1987; Alvarez et al., 2002). This could give rise to aberrant relationships between cortical thickness and D2/3 receptor availability both at baseline and in response to a *d*-amphetamine challenge. ADHD subjects and controls showed divergent associations with cortical thickness for both.

Little is known about relationships between striatal D2/3 receptor availability and cortical thickness in healthy controls. We did not detect significant associations in our previous studies, and the ones we detected here can only be considered trends. Woodward et al. (2009) reported positive associations between regional D2/3 availability and gray matter volume and density in both the midbrain and some cortical areas including those that emerged in our analyses. Based on Woodward et al. (2009) we would expect cortical gray matter volume and density to strongly correlate with cortical thickness, and hence to correlate with D2/3 availability in the same regions. However, it is difficult to extrapolate these predictions to associations with striatal D2/3 receptor availability. There is some evidence that cortical and striatal D2/3 receptor availability are inversely related (Zald et al., 2010), and, based on this, one might expect to find inverse associations between cortical thickness and striatal BP_ND_ in healthy individuals, consistent with the trends we report here. Positive associations in the ADHD subjects could then reflect an anomaly of cross-regulation of cortical and striatal D2/3 receptors.

The most prominent associations of CT with D2/3 binding in ADHD subjects were seen in the anterior cingulate and insula, key components of the salience network (Seeley et al., 2007; Menon, 2015). The salience network encompasses the anterior insula and dorsal anterior cingulate along with key subcortical components in the ventral striatum, substantia nigra, ventral tegmental area, and amygdala (Menon, 2015). It is involved in the detection of motivationally salient stimuli for adaptively guiding attention and goal-directed behavior. To this end, the salience network facilitates sustained processing via transient signals from the anterior insula that engage cognitive control systems (Menon and Uddin, 2010). The involvement of the salience network in task set maintenance manifests as sustained activation in fMRI experiments over entire blocks of trials (Dosenbach et al., 2008).

Difficulties with sustained attention and engagement are hallmarks of ADHD. Not surprisingly, ADHD subjects have been repeatedly found to have aberrant task-related activity in key regions of the salience network (Bush, 2010; Cortese et al., 2012; Plessen et al., 2016), as well as aberrant resting state salience network activity. Most are findings of hypo-connectivity of the salience network with other large scale networks, such as the default mode network, the central executive, dorsal attention, and sensory-motor networks (Carmona et al., 2015; Kucyi et al., 2015; Sidlauskaite et al., 2016) as well as hyper-connectivity within the network (Tian et al., 2006; McCarthy et al., 2013; Barber et al., 2015). This network imbalance may underlie the greater susceptibility of people with ADHD to being distracted by salient task-irrelevant material. The associations we saw with D2 receptor availability could suggest that DA-ergic abnormalities in ADHD could contribute to this network imbalance driving proneness to distraction. There is only indirect evidence linking DA transmission to salience network activity in ADHD: a recent meta-analysis found that stimulant medications, which modulate DA signaling, have their most consistent effects on cortical activity in inferior frontal/insula regions and dorsal anterior cingulate and adjacent regions (Rubia et al., 2014). Recently, suboptimal tonic DA signaling in the salience network was theorized to underlie reduced capacity to maintain a focused state, and suboptimal phasic signaling was posited to underlie impulsivity (Aboitiz et al., 2014). Our findings provide a more direct demonstration of a link between the DA system and the salience network in ADHD.

Though the association of baseline D2 binding with anterior insula thickness should be interpreted with caution due to its *post-hoc* nature, this finding could have implications for understanding emotional lability in ADHD. Emotional lability is increasingly recognized as an important contributor to functional outcomes (Shaw et al., 2014). Recently, a large study using an RDoC-based approach classified 437 children with and without ADHD into behavioral subtypes using temperament dimensions (Karalunas et al., 2014). Children belonging to the “irritable” subtype characterized by negative emotionality and poor emotion regulation showed weaker anti-correlations between amygdala and anterior insula resting state activity that other children, highlighting anterior insula as a potentially important neural substrate for emotional lability in ADHD. Our findings link this region to DA function in ADHD. Evidence of a relationship between striatal D2 receptor availability and the salience network's morphology does not necessarily reflect changes in function. Nonetheless, this possibility has some support. First, structural integrity of the salience network appears to be important for successful default mode network suppression (Bonnelle et al., 2012); more specifically, cortical thinning in this network predicted poorer performance on sustained executive control tasks in aging adults (Schmidt et al., 2015). Second, network dysfunctions in ADHD have been found to co-occur with structural deficits in network components (Kessler et al., 2014). Third, there have been several reports pointing to DA-dependent modulation of salience network connectivity, including DA genotype (DRD2 and COMT) effects on intrinsic salience network connectivity (Tian et al., 2013; Zhao et al., 2015) and reductions in intrinsic connectivity of this network resulting from acute *d*-amphetamine administration (Schrantee et al., 2015). Notably, reduced D2 binding in the salience network was associated with associative striatal DA depletion and executive dysfunction in Parkinson's disease (Christopher et al., 2014, 2015). Future studies combining the DA system imaging and functional imaging of the salience network could characterize the role of relationships between salience network and DA signaling abnormalities in ADHD.

Not all regions showing positive associations with D2/3 binding in the ADHD group belong to the salience network, and the meaning of associations in the cerebellum and cuneus is unclear. The cerebellum is thought to be closely linked with the salience network (Dosenbach et al., 2008), and was reported to show hyper-connectivity with the salience network in ADHD subjects (Kucyi et al., 2015). Occipital areas interact with the dorsal attention network to maintain attention (Capotosto et al., 2009; Shulman et al., 2009) and may be indirectly linked with the salience network and perhaps aberrantly so in ADHD. The occipital cortex has been implicated in the pathophysiology of ADHD in resting networks and in large longitudinal functional and structural imaging studies (Proal et al., 2011; Castellanos and Proal, 2012; Franke, 2016).

In conclusion, we found that stimulant-naïve ADHD adults did not show the negative association between amphetamine-induced changes in D2/3 receptor availability and prefrontal cortical thickness that we previously found in healthy controls (Casey et al., 2013; Jaworska et al., 2017). To the contrary, the associations were in the opposite direction. We also found evidence of anomalous associations between striatal D2/3 receptor availability and cortical thickness in components of the salience network. Evidence of morphological, functional, and neurotransmitter abnormalities in ADHD continues to grow, yet the possible relationships between these abnormalities remain largely uninvestigated. Although the results reported here are largely exploratory and derived from a small sample, which is a major limitation, they are a first step toward characterizing these relationships and point to a need for further study. Because studies in small samples can yield false positive findings, it would be important for our findings to be replicated using larger samples. Given the availability of structural MRI data from participants in many PET studies, future studies and meta-analyses of the existing datasets could evaluate the replicability of the current findings and further investigate the relationship between cortical morphology and both cortical and striatal DA in ADHD with only modest extra investment.

## Author contributions

MC carried out the study, analyzed the data, and prepared the manuscript; NF recruited and tested four of the control and five of the ADHD subjects; KC recruited and tested four of the control subjects; KL contributed to data analysis; GO contributed to data analysis and manuscript preparation; LH and RJ ascertained the ADHD diagnosis for the ADHD subjects; GB was responsible for determination of plasma amphetamine concentrations; AE provided critical input on interpretation of cortical thickness relationships. AD participated in study design; JP participated in subject recruitment and testing; CB contributed to the study design, supervised data collection, and acted as the supervising physician during testing; ML conceived of the study, supervised data collection, and recruited and tested three control participants; ML and CB had full access to all of the data and take responsibility for the data integrity and accuracy of analyses. All authors discussed the results and commented on the manuscript.

### Conflict of interest statement

The authors declare that the research was conducted in the absence of any commercial or financial relationships that could be construed as a potential conflict of interest.
